# Belly Dancer's Dyskinesia: A Glimpse of a Rare Phenomenon

**DOI:** 10.7759/cureus.1457

**Published:** 2017-07-11

**Authors:** ASHUTOSH GUPTA, Suman Kushwaha

**Affiliations:** 1 Neurology, Institute of Human Behavior and Allied Sciences, Delhi

**Keywords:** belly dancer, dyskinesia, domperidone

## Abstract

Belly dancer's dyskinesia (BDD) is an extremely rare manifestation consisting of involuntary and repetitive rhythmic movements of the abdominal wall. These movements cannot be voluntarily suppressed but may be influenced by respiratory maneuvers. Investigations such as spinal cord and abdominal imaging usually fail to reveal any local abnormalities to explain the movement disorder.

A 23-year-old male presented with sudden onset of undulating movements of the abdominal wall for the last two months after he took domperidone. There was no associated pain or effect of respiration. The movements used to subside during sleep. His radiological and hematological evaluations were inconclusive. The movements, however, subsided after administration of promethazine and clonazepam.

The cause of BDD varies, making diagnosis difficult. One of the causes being drug induced but it has never been reported earlier by domperidone. Also, our report provides a possible way to manage BDD by clonazepam and promethazine.

## Introduction

Dyskinesia limited to the axial musculature is an extremely rare phenomenon [1]. When such a dyskinesia involves the anterior abdominal wall, it is termed as belly dancers dyskinesia. The clinical characteristics of this unusual dyskinesia are somewhat variable but usually consists of involuntary, repetitive, sometimes painful and often rhythmic movements of the anterior abdominal wall with the majority being bilateral having slow writhing. Various cases have been reported in the literature with a long list of underlying causes. However, the exact underlying pathophysiology has not been elucidated. In such cases, symptomatic management is the only available option. Recovery is incomplete in most of these cases. Informed consent statement was obtained for this study.

## Case presentation

A 23-year-old male presented to the Movement Disorder Clinic of Institute of Human Behaviour and Allied Sciences, Delhi, India with the complaint of undulating movements of his anterior abdominal wall for the last two months. These movements were sudden in onset and non-progressive in nature. On detailed history, the patient had acute gastroenteritis at onset, for which he took antibiotics and domperidone for two days. These symptoms started three days after the medication.

The patient was admitted to the neurology ward for indoor observation, detailed examination and evaluation. These abdominal movements were fast, continuous and voluntarily uncontrollable. He was examined both in supine and sitting position respectively (Videos 1-2). There was no associated pain or affect of normal respiration on these movements. The movements were reduced in number on maneuvers like deep inspiration, breath-holding or counting numbers but still persisted. A video couldn’t be made at that moment. It was further observed that when the patient was in deep sleep, these movements used to subside.

**Video 1:** Belly dancer's dyskinesia with the patient in supine position Video showing the patient in supine position with involuntary, fast, repetitive movements over anterior abdominal wall –both abdominal wall protrusions and abdominal depressions without any other movement anywhere else in the body

**Video 2:** Belly dancer's dyskinesia with patient in sitting position Video showing the same patient in sitting position with similar movements over anterior abdominal wall. A prominent abdominal depression in supra-umbilical area is noted during contractions

**Figure 1:**
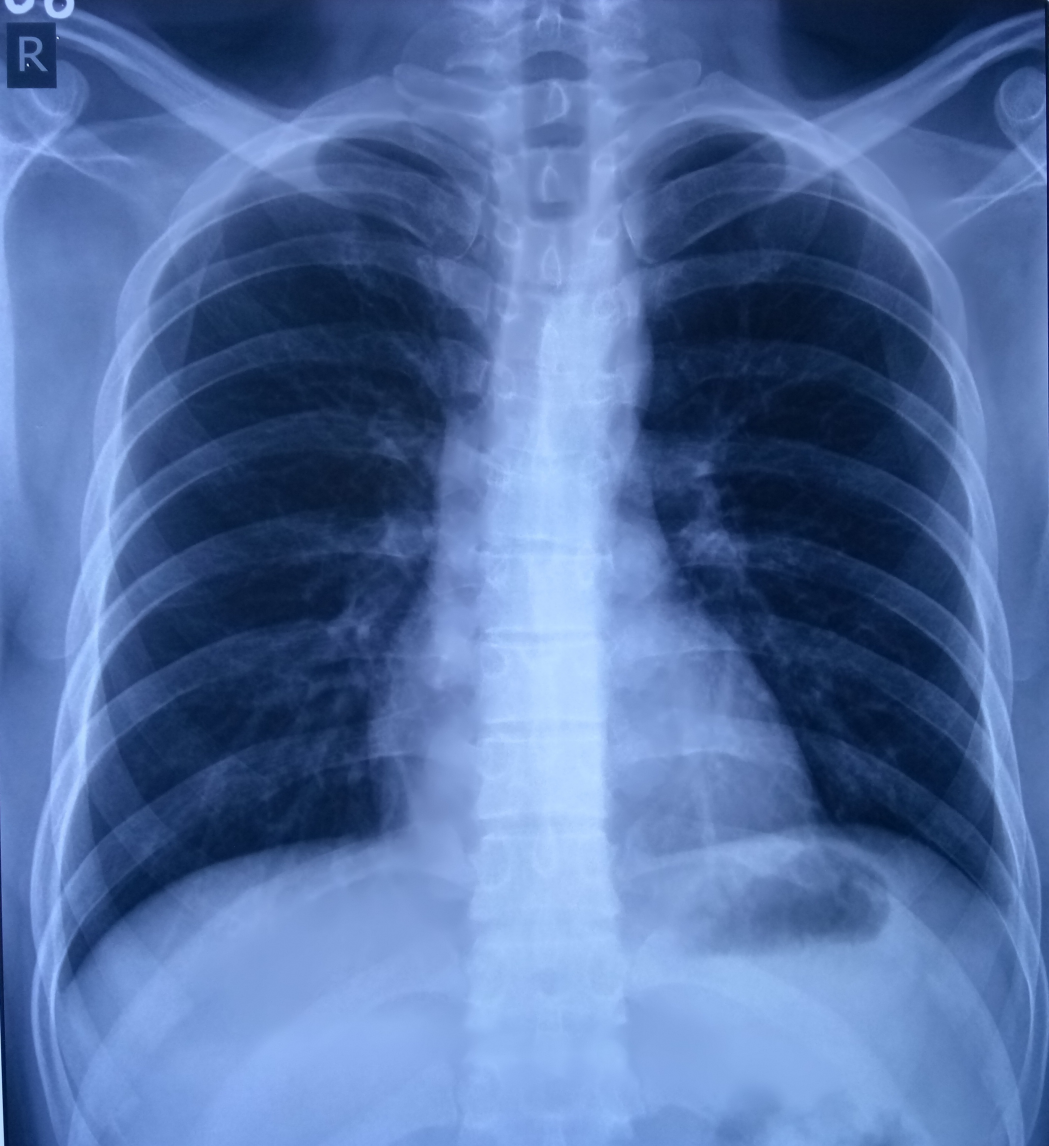
Chest X-Ray of the patient with belly dancer's dyskinesia showing apparently normal study

**Figure 2:**
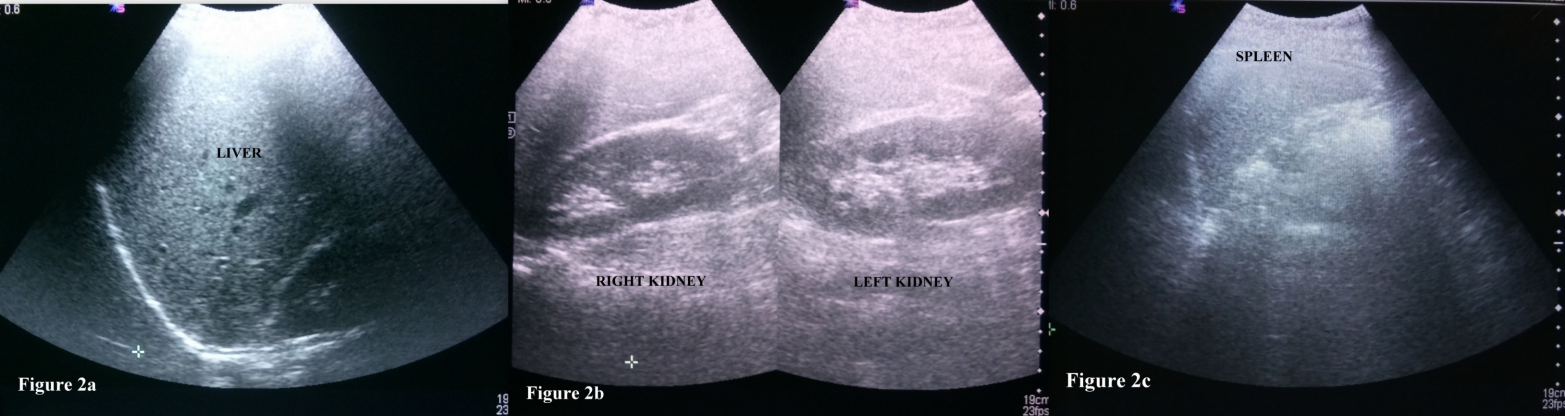
Ultrasound abdomen of the patient with belly dancer's dyskinesia Figure 2a showing normal liver size and echotexture. Figure 2b showing bilateral kidneys having normal size and preserved corticomedullary differentiation. Figure 2c showing spleen with normal size and shape

His brain and spinal cord imaging as well as routine blood investigations were negative for any abnormality or pathological conditions. Chest X-ray and ultrasound abdomen were also negative (Figure 1-2). Diaphragmatic fluoroscopy and abdominal wall/diaphragm electromyography was not done. He was prescribed injection promethazine with oral clonazepam. His symptoms subsided after two days of medication and never recurred.

## Discussion

Belly dancer’s dyskinesia is an extremely rare phenomenon described as various case reports in the literature, initially described by Iliceto G, et al. [2]. A thorough patient history and careful examination are necessary to obtain a correct diagnosis of BDD. Various modalities have been proposed for diagnoses and treatment, discussed below, but the management of this condition is extremely difficult and challenging [1].

The clinical manifestations of BDD include fluttering or rolling of anterior abdominal wall. The movements in BDD are involuntary, repetitive, semi-continuous, sometimes painful, often rhythmic and slow writhing [3]. The movements present in our case were faster in amplitude and frequency than the usually reported cases. Such types of movements are not suppressed on breath holding or distraction but may subside during sleep. Patients may also present with shortness of breath, chest pain or fatigue [4].

The onset of BDD is usually gradual and the movements are due to the variable combination of contractions of the rectus abdominis, oblique muscles, paraspinal, and perineal muscles. The pathophysiology of this condition is still unclear. It has been postulated that these abnormal movements are due to a dysfunction of inhibitory spinal interneurons or structural reorganization of local neuronal circuits [2].

BDD has a long list of causes spanning a gamut from post-operative to tumor (Table-1). When the syndrome is caused by irritation of the phrenic nerve, the heart is the most common source of irritation. Such cases result in diaphragmatic flutter synchronous with the systole. When diaphragmatic flutter is present bilaterally, a central origin is more likely. BDD arising from a central origin are usually present during sleep whereas the movements originating from peripheral or spinal origin invariably would subside [5]. Psychogenic factors are also frequently suspected in such cases as these symptoms only present while the patient is awaken [6]. Distractibility and breath holding may serve the purpose of diagnosis in such a dilemma. A history of recent surgery or trauma should be sought while evaluating BDD.

**Table 1:** Table showing the various etiologies of abdominal wall dyskinesia

Post abdominal surgery Uncomplicated vaginal delivery Osmotic demyelination syndrome Intramedullary thoracic cord tumor Tardive syndromes Compressive thoracic radiculopathy Levodopa-induced movements Diaphragmatic flutter Basal Ganglia lesions Functional movement disorders

Certain pharmacological agents were found to induce the BDD. In one case, an 80-year-old male developed diaphragmatic flutter after beginning a course of galantamine for the treatment of Alzheimer's disease. This medication was identified as the source of his affliction upon cessation of medication [7]. Linazasoro, et al. reported another instance of BDD being induced by the patient’s chronic use of clebopride, a drug utilized in the treatment of digestive disorders [3]. But BDD has never been reported with domperidone, as it probably occurred in our case.

Diagnosing BDD is mainly clinical but may be supplemented by fluoroscopy and electromyography. But as the tests are not being standardized, using them for diagnosis is still debatable. Brain and spinal cord imaging, in suspected cases be utilized to rule out the secondary causes.

No clinical studies have addressed the treatment options of BDD. Current treatment relies on expert opinion and case reports. Several pharmaceuticals have been utilized in the treatment of BDD. These are mainly of cosmetic concern and are difficult to treat despite numerous pharmacological agents. Diphenylhydantoin is frequently used to treat this disorder, but it is not effective in all the cases of BDD [6].

Diazepam has also shown to be beneficial in treatment of BDD. Haloperidol has also been tried successfully in one case [6]. Clonazepam has been shown to reduce both the frequency and amplitude of the abdominal contractions associated with this disorder and in some cases, completely reverse the symptoms of diaphragmatic flutter. However, one report described a case where the cessation of clonazepam therapy was directly followed by a return and progressive worsening of the syndrome [8]. This is in contrast to our study, where the symptoms subsided after stopping clonazepam and never recurred. Aripiprazole has been successfully tried in treatment of tardive diaphragmatic flutter in an elderly man reported by YH Chen, et al. [9].

When pharmacologic therapies prove ineffective, phrenic nerve block or crushing can alleviate symptoms of unilateral diaphragmatic flutter. Relief of symptoms is instantaneous. It can be ascertained that the intact phrenic nerve, enabling the full function of one hemisphere of the diaphragm, can maintain oxygenation at adequate levels during the patient undergoing either rest or exercise. Interestingly, after diaphragmatic function returns to normal, the symptoms of BDD do not return [4]. Recently, ultrasound guided botulinum toxin A injections has been given with success to a cohort of patients, after an unsatisfactory course of medical treatment. So far, no treatment modality has been recommended for the management of this condition. Therefore, the prognosis of BDD is highly unpredictable [10].

Although the horizon of both symptomatology and treatment is expanding, success is not a guarantee in afflicted patients. In summary, due to the varying causes of BDD, many methods of treatments have been tried. Our case provides a possible way to manage the BDD.

## Conclusions

Belly dancer's dyskinesia (BDD) is difficult to diagnose and often refractory to medical therapy. The exact mechanism is inconclusive. It may also be induced following administration of domperidone. Administration of oral clonazepam and injectable promethazine is suggestive to control the symptoms of BDD.
